# Menstrual characteristics, disorders and associated risk factors among female international students in Zhejiang Province, China: a cross-sectional survey

**DOI:** 10.1186/s12905-019-0730-5

**Published:** 2019-02-18

**Authors:** Emmanuel Ansong, Samuel Kofi Arhin, Yaoyao Cai, Xinxin Xu, Xueqing Wu

**Affiliations:** 1 Department of Obstetrics and Gynaecology, The First Affiliated Hospital of Wenzhou Medical University, Nanbaixiang, Street, Ouhai District, Wenzhou, Zhejiang Province 325000 People’s Republic of China; 2 Department of Reproductive Medicine, The Second Affiliated Hospital of Wenzhou Medical University, Xueyuan Western Road, Lucheng District, Wenzhou, Zhejiang Province 325027 People’s Republic of China

**Keywords:** Menstruation, Menarche, Premenstrual symptoms, Dysmenorrhea, International students

## Abstract

**Background:**

Until now, no previous study has addressed the menstrual patterns among female international students in China. In this present study, our objectives are to ascertain the menstrual characteristics and address the menstrual problems together with their associated risk factors among international students in China.

**Methods:**

A cross-sectional survey was carried out with 500 previously piloted self-structured questionnaires which were administered to female international students enrolled in 15 universities in Zhejiang Province, China from May 2–August 31, 2016. Participants were required to provide answers to a semi-structured questionnaire which asked questions about their socio-demographics and their menstrual characteristics while in their home countries. Relevant changes in their menstrual pattern in terms of amount of flow and duration, regularity, length and suggestive symptoms for premenstrual syndrome in the months after coming to reside in China were also inquired. Respondents indicated changes in eating habits and selected stressors relevant to them from a given list. Their stress levels were assessed and further categorized with the help of the Perceived Stress Scale (PSS). Measurements for the main outcomes included the characteristics of menstrual patterns after arrival in China, stress levels, stressors and new lifestyle patterns.

**Results:**

Four hundred and nine (81.8%) out of the 500 questionnaires met the criteria and constituted the population for this study. The respondents’ mean age was 21.405 years. Almost half of our respondents (49.1%) reported varying changes in their menstrual pattern after arrival to China. Although, menstrual regularity, normal menstrual length (21-35 days) and duration of flow (3–7 days) remained fairly normal among most of the respondents, disorders like premenstrual symptoms (PMS) (33.82%); abnormal amount (17.97%) and dysmenorrhea (16.38%) were prevalent. There was a significant association between high stress (PSS > 20) and menstrual change 0R = 1.636, 95% CI 1.051–2.547, *p* = 0.029) and dysmenorhea (*p* = 0.037). Common stressors included language barrier 81(25.88%), food 64(20.45%), and loneliness 56(17.89%).

**Conclusion:**

Menstrual disorders are high among international students in China. We established premenstrual symptoms as the most common menstrual disorder. High levels of stress (PSS > 20) emanating from factors including the language barrier, diet and loneliness was significantly related to the disruptions in their menstruation.

## Background

China has become one of the popular study hubs for international students as a result of the recent call for the internationalization and globalization of our world [1]. In 2015 alone, China’s Ministry of Education reported a total of 397,635 overseas students enrolled in various institutions. This quotation was almost 6% higher than that of 2014, which recorded a total of 377,054 students. The top three famous study destinations for these inbound students were Beijing, Shanghai and Zhejiang [2]. Although the statistics do not specifically detail the number of female students, we believe they constitute a larger portion of this international community.

Menstruation is a normal physiological phenomenon among women of reproductive age. Disorders that are usually linked with menstruation affect women from all parts of the world and it’s increasingly becoming one of the major reasons for gynecological visits. Menstrual irregularity is associated with prolonged menstrual bleeding and usually occurs right after the age of menarche as a result of annovulatory cycles. Instability in the endometrial lining that arises as a result of the uncontrolled production of unopposed estrogen causes the breakdown in vasoconstriction and myocardial contractility [3]. During the ovulatory cycle, the production of the prostaglandins in excess is thought to be the cause of the pain in dysmenorrhea. The presence of the prostaglandins causes the contraction of the myometrium and vasoconstriction locally. Elevated levels of serum vasopressin, nitric oxide and interleukin-6 have also been reported to be implicated in dysmenorrhea [4–6].

Among the student population, disturbances arising from menstruation could contribute to absenteeism, exemption from physical exercises and social and emotional distress. Among adolescents however, delayed, irregular, painful and heavy menstruation are extremely common and remain the chief reasons for physician visits [7]. Premenstrual syndrome greatly affects the student population and causes a considerable amount of anxiety [8]. Dysmenorrhea and menstrual irregularity are also reportedly prevalent among the student population and these also affect these young women’s social life and class attendance [5, 9, 10]. On the whole, students who are victims of menstrual disturbances also experience a tremendous impact on their social and physical health [11]. Apart from the pathological factors that disrupt women’s cycles, certain environmental factors and dietary or lifestyle trends have also been reported to influence the menstrual pattern like depression, cigarette smoking, changes in body weight and stress [12–15]. International students are reported to be exposed to a wide range of factors in their new environment and are likely to develop new lifestyle habits to help them cope and adapt to their new environment [16–18].

The paucity of information regarding menstrual patterns and the risk factors for menstrual irregularities among female international students in China make this current study extremely necessary. The main focus of the authors of this study was to identify the common menstrual disorders and the factors in their new environment that predispose them to menstrual disturbances. We hypothesized that menstrual changes among international students could be associated with the stress of having to cope or survive in their completely new environment.

## Methods

This cross-sectional survey was done from May to August, 2016 among female international students selected from 15 universities located in major cities like Hangzhou, Ningbo and Wenzhou, all of which are in Zhejiang Province, China. Permission to carry on with this study was granted after careful evaluation by the Ethics Committee of The First Affiliated Hospital of Wenzhou Medical University in April 2016 (No.wyyy2016–011). Female students included in this study were enrolled in these selected universities, and had been living in China for a year. They had no prior history of primary amenorrhea, bleeding disorders, or any history of abdominal or pelvic surgery. Also, they were at the time of the study not on any medication that could influence their menstrual patterns. Data collection was done using a pretested, structured and validated self-administered English questionnaire (SAQ) consisting of rating scales and close ended questions. We clarified the aims of the study and students were assured that their participation in the study was absolutely voluntary. Prior to the dissemination of the questionnaires, eligible participants were offered instructions sheets on how to answer the questionnaires and had to finally offer an oral acknowledgement whether or not reading and understanding English language was problematic to them. Our research assistants obtained verbal consent from all participants before they proceeded to complete the questionnaires. Instructions were given to complete the questionnaires without inputting their names and were given confidentiality assurance. The questionnaires inquired facts about their socio-demographics, menstrual pattern characteristics and living conditions. We asked them to characterize their menstrual patterns before they arrived to China and also indicate any observable changes in terms of regularity of menstrual cycle, duration and length, amount of menstrual flow and nature of associated menstrual symptoms after their arrival to China. This was to provide us with the opportunity to properly classify the section of participants with changes in their menstrual pattern and to look for potential risk factors. The criteria that defined menstrual cycle were as follows; irregular menstrual cycles: varying cycle length less than 21 or more than 35 days, regular menstrual cycles: cycle length of 21–35 days, hypomenorrhea (menstrual flow less than 2 days), prolonged menstrual flow (menstrual flow of more than 7 days), and amount of blood loss as reflected by the number of sanitary towels or pads changed per day during menstruation categorized as little, moderate and heavy for 4 pads, 5–7 pads and a maximum of 8 pads per day respectively. Included in the questionnaire were list of symptoms indicative of premenstrual symptoms (PMS) and respondents were to select ones applicable to them.

They were also asked to provide answers to questions regarding their living conditions which included specifics like dietary habits, stress and common stressors. We integrated the Perceived Stress Scale (PSS) in the question to enable us evaluate their stress levels. To evaluate their stress levels, we incorporated the Perceived Stress Scale (PSS), which examined their thoughts and feelings during the previous month. The scale is made up of 5-point Likert response format (0–4). The sum of the responses indicates the total score. Overall scores on the PSS-10 can range from zero to forty (0–40). Respondents with PSS value of ≤20 and > 20 were categorized as low stress and high stress respectively. In addition, variables such as language barrier, academic pressure, money, loneliness, homesickness and food were provided for, which respondents could selectively indicate as stressors. New social habits including smoking, drinking, late sleeping, lack of exercise and lack of socialization were used to investigate new lifestyle patterns.

Where appropriate, data were expressed as numbers in the form of percentages or as mean ± SD. Data were taken through revision, coded and tabulated using the frequency and percentage to analyze and interpret the results. Logistic regression and odds ratio were done to identify risk factors for menstrual change and to test the significance of the variables, *p* < 0.05. All calculations were statistically carried out with the help of Statistical Package for the Social Science (SPSS); SPSS Inc., Chicago, IL, USA) version 20.0 for Microsoft Windows.

## Results

We circulated five hundred (500) questionnaires in total but 40 of them were not returned. Out of the 460 that were returned, 51 were rejected due to lack of information giving a response rate of 81.8%. The remaining four hundred and nine (409) questionnaires met the criteria and were used for the analysis. Two hundred and one (*n* = 201, 49.14%) respondents reported noticeable menstrual changes after arrival to China and were categorized as the “Change group”. Among two hundred and eight (*n* = 208, 50.86%) participants, menstrual patterns remained unchanged in the months after arrival to China and was referred to as the “No change group”.

### General characteristics and demographics of participants

The population had a median (± SD) of 27.89 ± 2.77 kg/m^2^ for BMI and a mean age of 21.405 years for a range of 17–35 years. Health Science 238 (58.19%) was the most popular program among the respondents. Only 49(11.98%) of the respondents read Business related courses with only 32 (7.82%) studying engineering courses. The larger portion of the respondents 203(49.63%) were nationals from Africa, with the least 20(4.89%) from Europe (Table 1).

**Table 1:** General Characteristics and Demographics of Participants. (*n* = 409)

DEMOGRAPHICS		STUDENTS POPULATION				
		CHANGE		NO CHANGE		*P* value
		Number	Percentage (%)	Number	Percentage (%)	
Age	17–20	87	43.28	80	38.46	
	21–25	100	49.75	120	57.69	0.239
	26–30	6	2.99	2	0.96	
	31–35	8	3.98	6	2.89	
BMI	Underweight(< 18.5)	1	0.50	1	0.48	
	Normal weight (18.5–24.9)	72	35.82	93	44.71	0.142
	Overweight (25–29.9)	77	38.31	78	37.50	
	Obese(> 30)	51	25.37	36	17.31	
Program	Health Science	118	58.7	120	57.70	
	Engineering	10	5.0	22	10.60	0.145
	Business	28	13.9	21	10.10	
	Others	45	22.4	45	21.60	
Country	Africa	110	54.72	93	44.71	
	Asia	60	29.85	87	41.83	0.024
	Europe	9	4.48	11	5.29	
	Arab	13	6.47	15	7.21	
	Others	9	4.48	2	0.96	

### Menstrual characteristics for the change group

Two hundred and one 201(49.14%) respondents reported varying changes in their menstruation (Change group). With a range of 9 to 16 years, majority 173 (86.07%) of the respondents had menstruated by age 13 with the mean onset of menarche as 11.830 years. Characteristic menstruation comprised: regular menses in 75.62% (152), 43.78% (88) for cycle length with a range of 21 to 35 days, 46.26% (68) for duration of flow with a range of 3–7 days and moderate flow as reflected by the use of 5-7pads/day was 32.34% (*n* = 65). Menstrual irregularity was recorded in 49(24.38%) of the respondents. PMS was the most prevalent 256 (33.82%) menstrual disorder among our respondents. Most physical premenstrual symptoms were headaches 66(22.07%), bloating 23(7.69%), breast tenderness 13(4.35%) and back pain 12(4.01%) with irritability 16(5.35%) scoring the most reported psycho-behavioral premenstrual symptom. Other reported abnormalities included; abnormal amount 136(17.97%), dysmenorrhea 124(16.38%), abnormal length 113(14.93%) and abnormal duration 79(10.45%) (Table 2).

**Table 2:** Menstrual Characteristics for the Change Group (*n* = 201).

Menstrual Characteristics	Parameters	Number	Percentage (%)
Age of menarche	9–10 years	33	16.42
	11–13 years	140	69.65
	14–16 years	28	13.93
	Total:	201	100.00
Menstrual regularity	Regular	152	75.62
	Irregular	49	24.38
	Total:	201	100.00
Menstrual Length	< 21 days	52	25.87
	21–35 days	88	43.78
	> 35 days	61	30.35
	Total:	201	100.00
Duration of flow	< 3 days	36	24.49
	3–7 days	68	46.26
	> 8 days	43	29.25
	Total:	147	100.00
Amount of flow	Little (4pads/day)	60	29.85
	Moderate (5–7 pads/day)	65	32.34
	Heavy (max 8pads/days)	76	37.81
	Total:	201	100.00
Dysmenorrhea (Abdominal cramps)		124	100.00
Premenstrual Symptoms(PMS)	Bloating	23	7.69
	Back Pain	12	4.01
	Headache	66	22.07
	Nausea & Vomiting	2	0.67
	Breast tenderness	13	4.35
	Constipation	0	0
	Irritability	16	5.35
	Fever	0	0
	Diarrhea	0	0
	Others	1	0.33
	None	39	13.04
	Total:	295	100.00

### Analysis of statistical significance between factors and menstrual changes

Among our overall population, 248(60.64%) and 161(39.36%) were classified as high stress (PSS > 20) and low stress (PSS ≤ 20) respectively according to the PSS scale. High stress (PSS > 20) was the significant factor among the population that showed a strong association between the two groups [*p* = 0.029, OR = 1.636,CI 1.021–2.547]. Alcohol consumption, lack of exercise and lack of socialization showed increased risks for menstrual change with (OR 1.993,CI 0.671–5.921), (OR 1.389 CI 0.891–2.167) and (OR 1.306 CI 0.562–3.038) respectively. Lack of exercise was credited as the prevalent 203 (49.63%) of new social habits developed after arrival in China. Late sleeping and lack of socialization were the next common habits among the respondents scoring 180(44.01%) and 29(7.09%) respectively. Only18 (4.4%) of the respondents had developed the habit of alcohol consumption (Table 3).

**Table 3:** Analysis of statistical significance between factors and menstrual changes(*n* = 409)

Indicator	Menstrual Pattern							
	No Change		Change		*P*-Value	Odds Ratio	OR95%CI	
	Number	%	Number	%			LOWER	UPPER
Age								
17–20	80	47.90	87	52.10	0.8676	1.1040	0.344752	3.535526
21–25	120	54.55	100	45.45	0.8548	0.8970	0.280026	2.873634
26–30	2	25.00	6	75.00	0.3408	2.6660	0.354385	20.05635
31–35	6	42.86	8	57.14	–	–	–	–
AOM								
9–10 years	27	45.00	33	55.00	–	–	–	–
11-13 years	155	52.54	140	47.46	0.8452	0.9406	0.508649	1.739472
14-16 years	26	48.15	28	51.85	0.4028	1.4368	0.614638	3.358937
BMI								
Normal	94	56.97	71	43.03	0.9886	4.2651E-9	0.0E0	.
Overweight	78	50.32	77	49.68	0.9999	10.4435	0.0E0	.
Obese	36	41.38	51	58.62	0.9996	7.7275E-9	0.0E0	.
Country								
Africa	93	45.81	110	54.19	0.1248	0.27794	0.054173	1.426003
Arab	15	53.57	13	46.43	0.1708	0.28153	0.045896	1.726932
Asia	87	59.18	60	40.82	0.0386	0.17774	0.034559	0.914166
Europe	11	55.00	9	45.00	0.0470	0.15242	0.023799	0.976166
Others	2	18.18	9	81.82	–	–	–	–
Program								
Health	120	50.42	118	49.58	0.4653	0.773	0.387	1.543
Business	30	42.86	40	57.14	0.898	1.053	0.478	2.317
Engineering	32	64.00	18	36.00	0.028	0.367	0.150	0.895
Others	26	50.98	25	49.02	–	–	–	–
Stress								
≤ 20	96	59.63	65	40.37	–	–	–	–
> 20	112	45.16	136	54.84	0.029	1.636	1.051	2.547
Food								
Chinese	37	48.68	39	51.32	0.693	0.897	0.523	1.539
Western	154	50.00	154	50.00	–	–	–	–
CAW	17	68.00	8	32.00	0.158	0.523	0.213	1.284
Social								
Drinking	7	38.89	11	61.11	0.215	1.993	0.671	5.921
Habits								
LS	101	56.11	79	43.89	0.254	0.766	0.485	1.211
LOE	94	46.77	109	53.23	0.147	1.389	0.891	2.167
LOS	13	44.83	16	55.17	0.535	1.306	0.562	3.038

### Main stressors among the general population

**Fig. 1:**
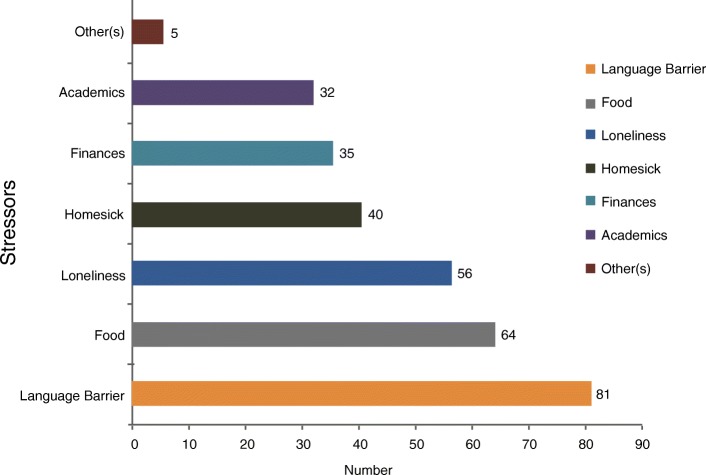
Main stressors among the general population

The commonest stressor among the study population was the language barrier 81 (25.88%) followed by food 64(20.45%), loneliness 56(17.89%) and homesickness 40(12.78%). Finance was considered as a stressor among 35(11.18%) with only 32(10.22%) rating their respective academic programs as a form of stressor (Fig.1).

### Relationship between high stress level and menstrual disorders

We attempted to establish the relationship of the various factors with menstrual irregularities and found out high stress was also significantly associated the risk of dysmenorrhea (*p* = 0.037). Although other factors like age, age of menarche, program of study, alcohol consumption, lack of exercise, lack of socialization and BMI did not show strong significant associations they increased the respondents’ risk of developing menstrual irregularities (Table 4).

**Table 4:** Relationship between high stress level and menstrual disorders (*n* = 201)

Menstrual Disorders	Grade				*P*-Value	Odds Ratio	OR95%CI	
	High stress		Low stress					
	Number	%	Number	%				
							LOWER	UPPER
Irregular	30	100.0	19	100.0	.892	.946	.425	2.105
Menstrual Length								
<21 days	35	44.30	17	50	.605	1.276	.506	3.218
>35 days	44	55.70	17	50	.453	.713	.295	1.724
Duration of flow								
<3 days	26	48.15	10	40	.118	.440	.157	1.233
>8 days	28	51.85	15	60	.871	.931	.390	2.220
Amount of flow								
Little	38	40.43	22	52.38	.156	1.976	.770	5.070
Heavy	56	59.57	20	47.62	.986	.992	.411	2.393
Dysmenorrhea	90	100.0	37	100.0	.037	.172	.033	.896
PMS	136	100.0	65	100.0	.069	.940	.880	1.005

## Discussion

This is the maiden study done to throw more light on the menstrual characteristics, disorders and associated risk factors among female international students in Zhejiang Province, China. This study revealed that international students are exposed to factors in varying degrees that can potentially cause disruptions in their menstrual patterns. These irregularities can have significant effects on these women’s life, health and work [15]. In close to 80% women with normal ovulation, the estimated blood loss on average is about 33.2 ml (10-84 ml), cycle length of 26–35 days, bleeding follows a 3–6 day period with a range of 2–12 days with the maximum flow on the second day [11]. The onset of menstruation is marked by menarche which is a very important indicator of sexual maturity in females. The mean age for the onset of menarche is reported differently worldwide and Africans are reported to have the earliest age of menarche probably attributed to their high body mass index [19, 20]. The age of menarche is also crucial since women who experience menarche much later have a higher risk for menstrual irregularities [21].

Unusually infrequent (oligomenorhea), unusually light (hypomenorrhea), heavy flow (menorrhagia), metrorrhagia, unusually painful (dysmenorrhea), premenstrual syndrome (PMS), absent or delayed menstruation (amenorrhea) and unusually frequent (polymenorrhea) are among the types of menstrual disorders women suffer and the number one reason for gynecologic visits [22, 23]. The reasons for irregularities in menstruation vary as women age and can occur due to a plethora of conditions ranging from hormonal imbalances, pregnancy, malignancies, infections, diseases, trauma to the use of certain medications [24–29]. Environmental factors and new lifestyle trends like caffeine consumption, physical activity, stress, smoking, age, weight gain and diet have also been closely correlated with menstrual disorders [30–35].

Premenstrual menstrual symptoms (PMS) was ascribed the main menstrual disorder (33.82%) among the group with change in their menstrual pattern. PMS is characterized by a multifarious pattern change in mood, behavior and physical symptoms invariably experienced during the premenstrual phase and resolve quickly before the onset of menstruation. It is reported that approximately 5% of women experience severe forms of PMS that distorts both their personal and social interactions [8]. Unlike in our study, which indicated headaches as the commonest premenstrual symptom, literature reports irritability and anger as the commonest and severe forms of PMS with an onset than earlier to other symptoms [36].

Abnormal amount 136 (17.97%) was reported as the second menstrual disorder among our respondents with menstrual changes. Personalized estimation of menstrual flow volume and hygiene coupled with different sanitary products on the market make the estimation of the accurate menstrual blood flow extremely difficult. Various quantification methods for menstrual blood loss estimation have been reported to provide unreliable and conflicting outcomes [37]. In our study, we employed the use of the traditional method, whereby women self-estimated their menstrual loss by counting the number of sanitary products used per day. This method is convenient and has been reported to correlate with the correct amount of menstrual blood loss [38]. Nevertheless, future studies could preferably make use of a more reliable approach to quantify menstrual blood loss since the amount of menstrual blood loss is a useful tool in the assessment of an individual’s productivity, general well-being and iron status [39, 40].

Dysmenorrhea was the third predominant menstrual disorder (16.38%) among our respondents. Although it is a common gynecological complaint among women in developing countries, it’s characterized by varied prevalence rate in literature. In one large study conducted among Australian teenagers for example, the prevalence was 93% and in another study among 664 Egyptian teenagers, the prevalence was reported as 80% [41, 42]. This difference has been linked to individual differences in pain threshold, cultural differences in the perception of pain and the absence of a standardized universal method to assess and grade dysmenorrhea [43].

One of the widely used instruments employed in the assessment of psychological stress among individuals with respect to varying situations over the past months is the perceived stress scale (PSS) [44]. With the help of the PSS, our general population was categorized as low stress (PSS ≤ 20) and high stress (PSS > 20) and we discovered more than half (60.64%) of the study population were high stress. This buttresses the point that international students experience a great deal of stress that emanates from difficulties with adjustment, support, perceived discrimination, homesickness, insecurity, anxiety, depression, language and academics [16–18].

The female reproductive system is very sensitive to physical and physiological stress. Although small doses of stress can motivate individuals to respond to changes, face challenges and complete tasks, high stress can have detrimental physical and psychological health effects. When the corticotrophin-releasing hormone system is activated as a physiological response to stress, menstrual function can be consequently disrupted [45]. The correlation of menstrual irregularities and high stress in our study is in line with the various publications that have registered menstrual changes in universities students studying demanding courses or individuals with competitive life style including severe exercises [35, 46, 47]. High stress (PSS > 20) was also found to be significantly correlated with dysmenorrhea (*p* = 0.037) in our study. Stress impairs the development of the follicle by inhibiting the release of follicle-stimulating hormone and luteinizing hormone. This may in turn alter progesterone synthesis and release which may affect the action of prostaglandin. Other stress related hormones like adrenaline and cortisol also can influence prostaglandin synthesis and/or binding in the myometrium and may explain the role of stress in dysmenorrhea. A positive association has also been reported between perceived stress and the risk of dysmenorrhea in both longitudinal and cross-sectional studies [23, 48–50]. The most prevalent stressor among our respondents was language barrier 81(25.88%) which may be attributed to the study of Chinese language as part of their academic curriculum or their limited scope of socialization due to having very few English speaking colleagues around them. The issue of language barrier goes beyond just being a stressor, as it can hinder physician consultation, hamper doctor–patients communication or result in poorer compliance and less patient satisfaction [16]. Other common stressors among our study population included food 64 (20.45%), loneliness 56 (17.89%), homesickness 40 (12.78%) and academic issues 32(10.22%).

In our attempt to investigate our respondents’ new lifestyle trends, lack of exercise was found to be the prevalent new social habit developed months after living in China. A significant association between lack of exercise and premenstrual symptoms has been reported in several studies. In one publication, aerobic exercises was evidenced as one of the options to help alleviate the symptoms of premenstrual symptoms [51]. Late sleeping was the second reported habit after arrival to China. This finding is not surprising, since the issue of sleep problems is significantly escalated during university, and among international students, due to problems with adjusting to new environment and dormitory settings [52]. The few reports of lack of socialization as a new social habit among our students could be attributed to the issue of language barrier which limits the scope of socialization and interpersonal interactions.

Our present study is limited in some areas that we will like to address. Firstly, the present study involved students who were enrolled in universities studying very demanding courses, which could obviously be viewed as potential stressors. Further comparative studies with foreigners who are not students or domestic students from other provinces are needed to generalize the results. Causality inference of the factors identified as being correlated with menstrual change is highly limited by virtue of our choice of study design. Prospective follow up studies are therefore needed to look for causality. Another limitation of our study lies in the type of instruments we utilized. Although, the application of the 20 or higher cut-off point to categorize women with high stress was recommended by Cohen et al. [44], recall bias and differences in individualized interpretation could be a possible limitation since stress levels were assessed by questions from the previous months. The reader should also remember that participants estimated their menstrual amount and duration by calculating the quantities of sanitary pads used or by the number of days of menstruation and blood loss. This approach is likely to also suffer varying degrees of individual variations. Future studies could employ the use of a more objective quantification like the menstrual pictogram [53].

## Conclusion

Menstrual disorders are prevalent among the international student community as a result of a wide range of factors that contribute to the irregularity of menstrual patterns. In the present study, the most common disorder these students experienced was premenstrual symptoms, followed by abnormal amount and dysmenorrhea. The most predominant premenstrual symptom was headaches, followed by bloating and irritability. The top three main forms of stressors among the general student population were language barrier, food and loneliness. These stressors accounted for a high level of stress (PSS > 20) among more than half of the students who have witnessed changes in their menstrual pattern and this high stress was significantly correlated with dysmenorrhea. Although, the formation of new social habits did not show strong association with menstrual disorders among the students body, it was striking to note that majority of these international students do not engage in exercises and sleep rather late. With the large scale movement of international students into Chinese Universities and Colleges, we believe additional studies with larger population size across major universities in China are needed to broadly assess this problem to help illuminate its prevalence. It is important that prospective studies which are done can include factors that can influence stress, such as cultural and religious background, social support and personality type, so that respective universities can develop and strategize appropriate interventions to help eradicate or minimize the effect of these potential factors that predispose these individuals to menstrual irregularities.

## Abbreviations

BMI: Body mass index
CAW: Chinese and Western
LOE: Lack of exercise
LOS: Lack of socialization
LS: Late sleeping
PMS: Premenstrual symptoms
PSS: Perceived Stress Scale
